# Training the Polarization in Integrated La_0.15_Bi_0.85_FeO_3_‐Based Devices

**DOI:** 10.1002/adma.202104688

**Published:** 2021-10-24

**Authors:** Marvin Müller, Yen‐Lin Huang, Saül Vélez, Ramamoorthy Ramesh, Manfred Fiebig, Morgan Trassin

**Affiliations:** ^1^ Department of Materials ETH Zurich Zurich 8093 Switzerland; ^2^ Department of Materials Science and Engineering University of California Berkeley CA 94720 USA; ^3^ Materials Sciences Division Lawrence Berkeley Laboratory Berkeley CA 94720 USA; ^4^ Condensed Matter Physics Center (IFIMAC) and Departamento de Física de la Materia Condensada Universidad Autónoma de Madrid Madrid E‐28049 Spain; ^5^ Department of Physics University of California Berkeley CA 94720 USA

**Keywords:** BiFeO _3_, magnetoelectrics, multiferroics, operando, optical second‐harmonic generation

## Abstract

The functionalities of BiFeO_3_‐based magnetoelectric multiferroic heterostructures rely on the controlled manipulation of their ferroelectric domains and of the corresponding net in‐plane polarization, as this aspect guides the voltage‐controlled magnetic switching. Chemical substitution has emerged as a key to push the energy dissipation of the BiFeO_3_ into the attojoule range but appears to result in a disordered domain configuration. Using non‐invasive optical second‐harmonic generation on heavily La‐substituted BiFeO_3_ films, it is shown that a weak net in‐plane polarization remains imprinted in the pristine films despite the apparent domain disorder. It is found that this ingrained net in‐plane polarization can be trained with out‐of‐plane electric fields compatible with applications. Operando studies on capacitor heterostructures treated in this way show the full restoration of the domain configuration of pristine BiFeO_3_ along with a giant net in‐plane polarization enhancement. Thus, the experiments reveal a surprising robustness of the net in‐plane polarization of BiFeO_3_ against chemical modification, an important criterion in ongoing attempts to integrate magnetoelectric materials into energy‐efficient devices.

## Graphical abstract

A persisting net in‐plane polarization in highly La‐substituted multiferroic BiFeO_3_ films is probed operando using non‐invasive optical second‐harmonic generation directly in prototypical magnetoelectric device architectures. A spontaneous domain structure reordering triggered by the first electrical cycles leads to an abrupt enhancement of this net in‐plane polarization.

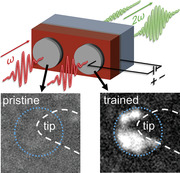

## 1 Introduction

The pursuit of non‐volatile and energy‐efficient data storage culminated recently in the proposal of magnetoelectric random‐access memory (ME‐RAM) and magnetoelectric spin‐orbit (MESO) logic devices.^[^ ^1^, ^2^, ^3^ ^]^ Magnetoelectric (ME) multiferroics (MFs), specifically materials exhibiting coexisting and coupled ferroelectric and ferromagnetic order, play a key role in the writing process. In these materials, a nearly dissipationless electric field can reverse the spontaneous magnetization as opposed to reversal by an electric current or a magnetic field, which would suffer from Ohmic losses and waste heat.^[^ ^3^, ^4^, ^5^ ^]^

BiFeO_3_ is one of the few known room‐temperature ME MFs.^[^ ^6^, ^7^ ^]^ Its functionality strongly depends on the ferroelectric domain structure. In weakly strained BiFeO_3_, a spontaneously formed ferroelectric stripe‐domain configuration facilitates a reliable reversal of the magnetic order by an out‐of‐plane electric field.^[^ ^1^, ^7^, ^8^ ^]^ When exchange‐coupled to a ferromagnetic layer, this translates directly to a reversal of the macroscopic net magnetization at room temperature.^[^ ^1^, ^8^ ^]^ At present, however, the technological merit of BiFeO_3_ is impeded by its large coercive electric field which exceeds the limit for technologically feasible operation by an order of magnitude.^[^ ^3^ ^]^

Chemically modifying BiFeO_3_ turned out to be a key to substantially reduce the coercive field.^[^ ^3^ ^]^ In particular, isovalent substitution of Bi with La has been shown to enable switching within the technological limits of a device.^[^ ^3^, ^9^, ^10^ ^]^ Ultimately, sub‐200‐mV electric‐field poling of a ferromagnetic state was demonstrated in Co_90_Fe_10_|La_0.15_Bi_0.85_FeO_3_ bilayers.^[^ ^3^, ^11^ ^]^

Such heavy chemical substitution perturbs the magnitude and direction of the spontaneous polarization as well as the structure and domain configuration of BiFeO_3_.^[^ ^10^, ^11^, ^12^ ^]^ The domain configuration in La‐substituted BiFeO_3_ was thus found to be highly randomized in comparison to pure BiFeO_3_. In addition, the crystal symmetry changes from rhombohedral to monoclinic.^[^ ^10^, ^11^, ^13^ ^]^ The loss of the regular BiFeO_3_ stripe‐domain structure is likely to impact characteristics relevant for application such as the aforementioned magnetoelectric switching process.

As the functionality of any ferroic is governed by the manipulation of its domains, non‐invasive operando studies on La‐substituted BiFeO_3_ integrated into a capacitor architecture are essential to understand the impact of the substitution‐induced domain disorder on the magnetoelectric switching behavior. Optical second‐harmonic generation (SHG) suggests itself for these investigations as it provides symmetry‐sensitive, non‐invasive, and time‐resolved access to a ferroelectric state even after integration of the latter into a device architecture.^[^ ^14^, ^15^, ^16^ ^]^

Here we show that, contrary to first impression, the domain configuration of BiFeO_3_ is not irrevocably compromised by the chemical modification. Despite the prevalent domain disorder in pristine La_0.15_Bi_0.85_FeO_3_, our SHG studies show a reminiscence of the polar order of pristine BiFeO_3_ in the form of a weak net in‐plane polarization. With the application of out‐of‐plane electric fields, we further trained this ingrained polarization to recover the pure‐BiFeO_3_‐like stripe‐domain configuration. Moreover, operando studies on La‐substituted BiFeO_3_ integrated into device‐like capacitor heterostructures revealed the same reorientation of the domain configuration after electric‐field training. Hence, our studies reveal an impressive, hidden robustness of the domain configuration of BiFeO_3_ against chemical modification, an important characteristic in tuning the performance of BiFeO_3_ toward usability in marketable oxide‐electronic devices.

## 2 Results and Discussion

Epitaxial BiFeO_3_ and La_0.15_Bi_0.85_FeO_3_ films with a thickness of 100 nm were grown on SrRuO_3_‐buffered (110)_o_‐oriented single‐crystalline DyScO_3_ substrates using pulsed laser deposition (see Experimental Section). Here, “o” refers to the orthorhombic lattice of DyScO_3_. The La substitution reduces the coercive field by pushing the material closer to the ferroelectric‐paraelectric phase boundary.^[^ ^10^, ^11^, ^17^ ^]^ The conducting SrRuO_3_ serves as bottom electrode and provides the electrostatic boundary conditions for the formation of stripe‐like 71° domains.^[^ ^14^, ^18^, ^19^ ^]^ An array of circular Pt|Co_90_Fe_10_ top electrodes with a thickness of 2.5 nm for both the Pt capping and the ferromagnetic layer completed the assembly of the Co_90_Fe_10_|La_0.15_Bi_0.85_FeO_3_|SrRuO_3_ capacitors. Electrode diameters of 20, 50, and 200 μm were used.

**Figure 1:**
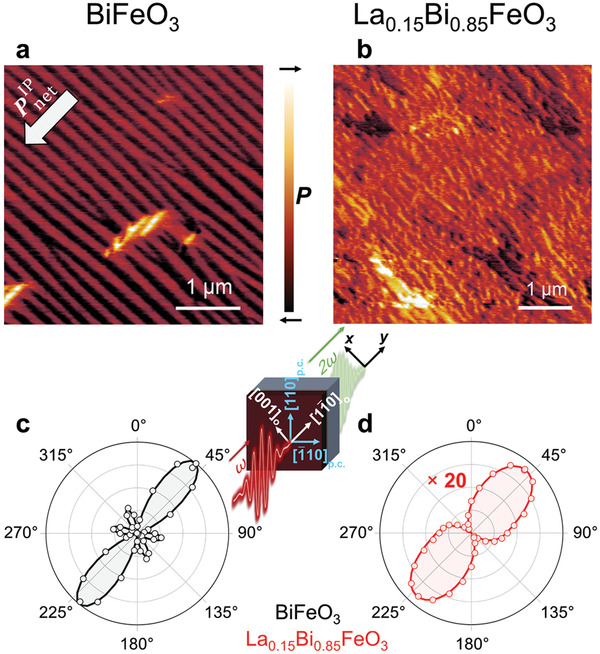
a) Lateral‐PFM image of as‐grown (001)_p.c._‐oriented BiFeO_3_ films of 100 nm. The index “p.c.” refers to the pseudocubic unit cell of BiFeO_3_. The white arrow depicts the net in‐plane polarization $P_{\text{net}}^{\text{IP}}$. b) Lateral‐PFM image of (001)_p.c._‐oriented La_0.15_Bi_0.85_FeO_3_ films of 100 nm. c,d) SHG anisotropy measurements (see Experimental Section) for the samples in (a) and (b), respectively. The SHG yield in (d) was multiplied by a factor of 20. The same intensity scale was used for (c) and (d). The solid lines in (c) and (d) show SHG fits using the point‐group symmetry *m* for BiFeO_3_ and La_0.15_Bi_0.85_FeO_3_ with an additional non‐zero contribution of the $\chi_{xxx}^{\left(2\right)}$ component for the latter. The illustration between (c) and (d) shows a sketch of our experiment with the respective coordinate systems. The axes *x* and *y* correspond to the monoclinic symmetry of the stripe domain structure and, thus, describe the coordinate system of our SHG fits.

In order to evaluate the impact of the La substitution on the pristine ferroelectric state, we begin our investigation by comparing the microscopic domain configuration of our BiFeO_3_ and La_0.15_Bi_0.85_FeO_3_ films using piezoresponse force microscopy (PFM). For BiFeO_3_, in agreement with the literature, we observe a periodic configuration of 71° stripe domains due to the anisotropic in‐plane strain induced by the (110)_o_‐oriented DyScO_3_ substrate, see **Figure** **1**a.^[^ ^14^, ^18^, ^19^, ^20^ ^]^ Note that the small lattice mismatch imposed by the substrate has negligible impact on the strain‐induced monoclinic distortion on the 71° domain wall.^[^ ^14^, ^19^, ^21^, ^22^ ^]^ This stripe‐domain structure yields a macroscopic net in‐plane polarization $P_{\text{net}}^{\text{IP}}$ which points along ${\left[1\bar{1}0\right]}_{o}$, that is, perpendicular to the domain walls.

In contrast, 15% La substitution has been shown to reduce the structural symmetry to monoclinic which gives rise to additional polarization domain states through reorientation of the polarization from 〈111〉_p.c._, as in BiFeO_3_, to 〈112〉_p.c._.^[^ ^10^ ^]^ Here, “p.c.” refers to the pseudocubic unit cell of BiFeO_3_ or La_0.15_Bi_0.85_FeO_3_. Despite the anisotropic in‐plane strain generated by the DyScO_3_ substrate, our lateral‐PFM scan depicted in Figure 1b shows isotropic domain disorder. In particular, the corresponding mosaic‐domain configuration exhibits domains with oppositely oriented in‐plane polarization components. This agrees well with the results of earlier studies and appears to cancel the net in‐plane polarization.^[^ ^10^, ^11^ ^]^

A precise, quantitative estimation of $P_{\text{net}}^{\text{IP}}$ in La_0.15_Bi_0.85_FeO_3_ by PFM is infeasible, however. Let us therefore compare the macroscopic polarization state of the pristine BiFeO_3_ and La_0.15_Bi_0.85_FeO_3_ thin films using SHG. This process denotes the frequency doubling of a light wave permitted as electric‐dipole‐type process in non‐centrosymmetric media. The strong correlation to the crystal symmetry and the relation between the SHG intensity ***I***_SHG_ and the spontaneous polarization ***P*** with ***I***_SHG_ ∝ | ***P*** |^2^ make SHG an excellent technique to verify the presence of a ferroelectric polarization background‐free and thus with outstanding sensitivity.^[^ ^23^, ^24^, ^25^ ^]^ Because of the spatial resolution of 3−4 μm, SHG probes the macroscopic polarization ***P***_net_ rather than the polarization of individual domains in our thin films. In other words, SHG allows us to surpass nanoscale probing methods such as PFM by coupling to the technologically relevant value of ***P***_net_ directly. Furthermore, SHG is only sensitive to the symmetry breaking in a plane perpendicular to the propagation vector of the incident light. Hence, we work in a normal‐incidence transmission configuration in order to single out the in‐plane component $P_{\text{net}}^{\text{IP}}$ of the net polarization, whereas unwanted SHG contributions from the out‐of‐plane polarization or from surfaces or interfaces are avoided.^[^ ^24^, ^26^, ^27^ ^]^ We provide a conceptual sketch of the experimental setup in Figure 1.

In Figure 1c,d we show the azimuthal anisotropy of the SHG signal (see Experimental Section) of as‐grown BiFeO_3_ and La_0.15_Bi_0.85_FeO_3_ films. For BiFeO_3_, we find a dominant double lobe along ${\left[1\bar{1}0\right]}_{o}$. As the macroscopic net polarization is the result of the superposition of two domain states with the same polarization magnitude and areal proportion, the stripe‐domain macro‐regions exhibit a mirror plane defined by the direction of the net polarization and the film‐surface normal. Our fit of the SHG contributions permitted for the resulting averaged point‐group symmetry *m* (see Experimental Section) is in good agreement with the measured SHG anisotropy, hence demonstrating the high quality and uniformity of our stripe‐domain configuration across the area probed with a laser‐spot diameter of ≈200 μm.

Quite unexpectedly, we also find a non‐zero SHG yield for the La‐substituted film which suggests a non‐vanishing macroscopic net polarization. This observation reveals a hitherto unrecognized non‐randomization in the domain configuration of the pristine La_0.15_Bi_0.85_FeO_3_ films. We obtain a quantitative estimate of the magnitude of $P_{\text{net}}^{\text{IP}}$ by calibrating the integrated SHG yield with respect to the SHG yield obtained from the perfect stripe‐domain configuration of BiFeO_3_ ($P_{\text{net}}^{\text{IP}}$ = 60.0 μC cm^−2^, see Supporting Information).^[^ ^28^, ^29^, ^30^, ^31^ ^]^ The drop in SHG yield in the La_0.15_Bi_0.85_FeO_3_ sample corresponds to $P_{\text{net}}^{\text{IP}}=12.8\;\mu C\;{\text{cm}}^{-2}$ = 12.8 μC cm^−2^ and, hence, to a remainder of $P_{\text{net}}^{\text{IP}}$ of 20% in comparison to the unsubstituted BiFeO_3_ films. The anisotropy of the La_0.15_Bi_0.85_FeO_3_ film further reveals a double lobe along ${\left[1\bar{1}0\right]}_{o}$, similar to BiFeO_3_. This indicates that $P_{\text{net}}^{\text{IP}}$ is oriented in the same crystallographic direction as in BiFeO_3_.

A closer comparison of Figure 1(d) with Figure 1(c) reveals the absence of minor lobes as well as an asymmetric shape with respect to the ${\left[1\bar{1}0\right]}_{o}$ direction in our La_0.15_Bi_0.85_FeO_3_ films. A fit of the anisotropy of the SHG signal from the La_0.15_Bi_0.85_FeO_3_ films in Figure 1(d) reveals that its angular dependence cannot be properly emulated with the SHG susceptibility components allowed for BiFeO_3_ in a 71° stripe‐domain configuration. We therefore expand our fit and lower the point‐group symmetry by introducing an additional $\chi_{xxx}^{\left(2\right)}$ component (see Experimental Section). This, ultimately, leads to a good agreement with our experiment.

Observation of $P_{\text{net}}^{\text{IP}}$ ≠ 0 suggests that La_0.15_Bi_0.85_FeO_3_ may be feasible for technological implementation after all. Let us therefore see whether we can gain control over $P_{\text{net}}^{\text{IP}}$ by electric‐field poling. We start by locally applying an out‐of‐plane electric field with a magnitude close to the saturation field using a biased scanning‐probe microscopy (SPM) tip to pole a region of 25 × 25 μm^2^ (see Experimental Section). We employ SHG microscopy to directly map the resulting magnitude and direction of $P_{\text{net}}^{\text{IP}}$.

**Figure 2:**
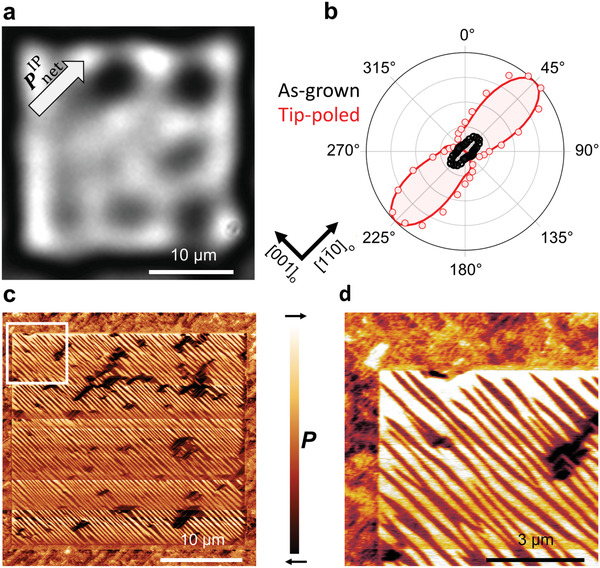
a) Spatially resolved SHG image of an electric‐field‐poled 25 × 25 μm^2^ square using a SPM tip on a (001)_p.c._‐oriented La_0.15_Bi_0.85_FeO_3_ film of 100 nm. The DC electric field applied was 700 kV cm^‐1^. b) SHG anisotropy on a poled (red) and as‐grown (black) region of the La_0.15_Bi_0.85_FeO_3_ film. The solid lines show corresponding SHG fits using the point‐group symmetry *m*. The fit for the as‐grown region requires an additional non‐zero contribution of the $\chi_{xxx}^{\left(2\right)}$ component. c) Lateral‐PFM image of the poled region in (a). d) Magnified view of the region outlined in (c).

Our SHG anisotropy measurements recorded on the poled and the pristine regions in **Figure** **2**b show that the orientation of $P_{\text{net}}^{\text{IP}}$ is not affected by the poling procedure. Strikingly, however, we observe a fivefold enhancement of the SHG yield in the switched area with respect to the surrounding pristine state when the optical configuration is set to probe the net polarization of La_0.15_Bi_0.85_FeO_3_ along ${\left[1\bar{1}0\right]}_{o}$, see Figure 2a. The corresponding increase of the net in‐plane polarization to 28.6 μC cm^−2^ suggests an electric‐field‐induced reorganization of the domain configuration. In addition, our symmetry analysis based on a fit of the SHG anisotropy of the poled region shows the restoration of the macroscopic point‐group symmetry *m*. For a detailed analysis of the nonlinear susceptibility tensor components, we refer to Table S1, Supporting Information. Our observations, consequently, suggest that the restoration of the stripe‐like domain configuration of BiFeO_3_ might, after all, be possible in La_0.15_Bi_0.85_FeO_3_.

In order to gain a spatially resolved image of the observed electric‐field‐induced reordering of the domain configuration in La_0.15_Bi_0.85_FeO_3_, we used PFM. A lateral‐PFM image of the poled region is depicted in Figure 2c with a magnifying scan of the outlined region shown in Figure 2d. The lateral‐PFM image indeed confirms the unprecedented recovery of the stripe‐like domain structure characteristic for BiFeO_3_ from the disordered domain configuration of as‐grown La_0.15_Bi_0.85_FeO_3_.

**Figure 3:**
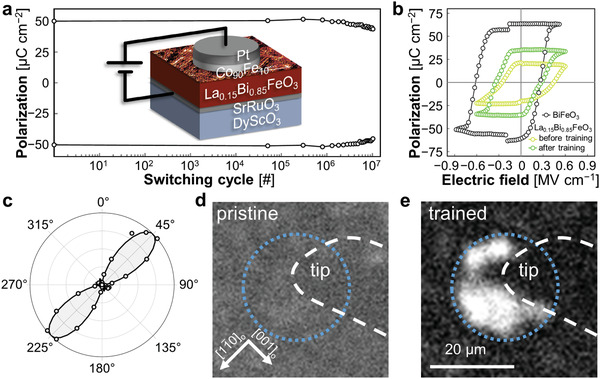
a) Characterization of the ferroelectric fatigue of a capacitor‐like Co_90_Fe_10_|La_0.15_Bi_0.85_FeO_3_|SrRuO_3_ heterostructure after electric‐field training. A sketch of the capacitor architecture is shown as inset. b) Ferroelectric hysteresis loops of pristine BiFeO_3_ and of La_0.15_Bi_0.85_FeO_3_ before and after the electric‐field training. The loops were measured with the positive‐up‐negative‐down (PUND) technique at room temperature (see Experimental Section). c) SHG anisotropy of the capacitor region after electric‐field training. The solid line shows the SHG fit using the point‐group symmetry *m*. d,e) SHG images of the capacitor before and after electric‐field training. The region outlined with the white dashed line marks the tip of our electric probes. The region encircled with the blue dotted line marks the location of our capacitor with a diameter of 20 μm.

The question remains as to what extent the observed restoration of $P_{\text{net}}^{\text{IP}}$ by electric‐field training can be sustained in real‐device operation. After all, the electric in‐plane trailing field caused by the moving tip in our experiment is not representative for the electric field of a capacitor‐like electrode|La_0.15_Bi_0.85_FeO_3_|electrode geometry.^[^ ^32^ ^]^ We therefore sandwich the La_0.15_Bi_0.85_FeO_3_ in between a SrRuO_3_ bottom and a Co_90_Fe_10_ top electrode (see Experimental Section). A schematic of this capacitor, which is reminiscent of the core of a ME‐RAM unit or a MESO interconnect, is depicted as inset in **Figure** **3**a.^[^ ^1^, ^2^, ^3^ ^]^

We first test the functionality and the performance of our capacitors by applying the electric‐field training and performing ferroelectric fatigue tests (Figure 3a). The training field was 600 kV cm^−1^ and the pulse duration as well as the relaxation time in between two voltage pulses were 100 μs.^[^ ^33^, ^34^ ^]^ We find reliable and persistent ferroelectric switching events with a degradation of the polarization of only 10% after 10^7^ cycles. The ferroelectric hysteresis loops measured on both BiFeO_3_ and La_0.15_Bi_0.85_FeO_3_ are shown in Figure 3b and confirm the La‐doping‐induced decrease of the coercive field. For the La_0.15_Bi_0.85_FeO_3_ films, we find an out‐of‐plane polarization with a magnitude of 25.0 μC cm^−2^, which is in excellent agreement with previous reports.^[^ ^10^, ^35^ ^]^

Now that we have demonstrated the reliability of our La_0.15_Bi_0.85_FeO_3_‐based capacitors, let us perform an operando study of the dynamics of the in‐plane polarization component which cannot be extracted from our fatigue test and hysteresis loop, shown in Figure 3a,b, respectively. For this, we exploit the capability of SHG to probe $P_{\text{net}}^{\text{IP}}$ of buried layers non‐invasively.^[^ ^15^ ^]^ In Figure 3d,e we show the spatially resolved SHG image of the capacitor heterostructure before and after the electric‐field training. The region outlined with a white dashed line marks the position of the tip of our electric probe. When we set the polarization of the ingoing and the emitted light to probe $P_{\text{net}}^{\text{IP}}$ along ${\left[1\bar{1}0\right]}_{o}$, we observe a strong increase in SHG yield after the electric‐field training. Note that this SHG enhancement is independent of the polarity of the poled state, see Figure S2, Supporting Information. Our analysis of the SHG anisotropy of the electrically trained capacitor device depicted in Figure 3c reveals that the intensity maximum and, thus, the axis of $P_{\text{net}}^{\text{IP}}$ remain along ${\left[1\bar{1}0\right]}_{o}$. We therefore conclude that the dynamics of the domains observed in the SPM tip‐poling experiments in Figure 2 is fully transferable to the device‐like architecture of the electrode|La_0.15_Bi_0.85_FeO_3_| electrode capacitor.

## 3 Conclusion

In summary, we have demonstrated an unexpected persistence of the BiFeO_3_‐like ferroelectric domain order in La_0.15_Bi_0.85_FeO_3_ thin films. Despite the apparent randomization of the ferroelectric domain configuration, we find a trace of the net in‐plane polarization of BiFeO_3_ imprinted in the La_0.15_Bi_0.85_FeO_3_ thin films. We use electric‐field training to largely restore the BiFeO_3_‐like stripe‐domain structure and the associated net in‐plane polarization. This behavior can even be transferred to device‐like architectures, which show a remarkable stability against ferroelectric fatigue up to 10^7^ switching cycles. Our results thus lay the foundation to achieve deterministic electric‐field control of magnetism in future ferromagnet|La_0.15_Bi_0.85_FeO_3_ thin film heterostructures, a vital technological asset that had been thought to be compromised by the La substitution.

## 4 Experimental Section

### Sample Preparation

The La_0.15_Bi_0.85_FeO_3_|SrRuO_3_ and BiFeO_3_|SrRuO_3_ films were grown on single‐crystalline (110)_o_‐oriented DyScO_3_ by pulsed laser deposition at 690–710 °C with a laser fluence of ≈1.2 J cm^−2^ and under a 100–160 mTorr oxygen pressure. They were cooled down to room temperature at 500 Torr oxygen pressure. After the cooling process, the films were transferred to the DC‐magnetron sputtering chamber at a base pressure of ≈1 × 10^−7^ Torr, that is, without breaking the vacuum. The top Pt (2.5 nm)/Co_90_Fe_10_ (2.5 nm) electrodes were deposited under an argon pressure ranging from 2 × 10^−3^ to 7 × 10^−3^ Torr. The electrodes were patterned by photolithography and argon plasma etching.

### Second‐Harmonic Generation Measurements

For the SHG measurements an amplified Ti:sapphire laser/optical parametric amplifier system emitting 130 fs pulses with a repetition rate of 1 kHz was used. For the experiments a fundamental wavelength of 1300 nm (0.95 eV) is chosen. The polarization of the incoming fundamental light beam was rotated using a half‐wave plate (polarizer). The polarization of the detected SHG light was selected with a Glan–Taylor prism (analyzer). The SHG light was detected with a liquid‐nitrogen‐cooled CCD camera. For the SHG microscopy experiments, a long‐working‐distance microscope objective was used to achieve a spatial resolution of 3−4 μm. All experiments were performed in a normal‐incidence geometry. The optical experiments were therefore sensitive to the ferroelectric in‐plane polarization component, while SHG contributions associated to the surface, interface or out‐of‐plane polarization were avoided.

Anisotropy scans were obtained by rotating the polarization of the fundamental light and of the projected polarization of the SHG light simultaneously in 10° steps while acquiring a SHG image at each step. The intensity of all pixels within a region of interest was summed up in each image to obtain the signal intensity which was then plotted against the orientation of the polarization of the fundamental light. The background signal originating from stray light as well as electronic noise in the detection process was subtracted.

### Second‐Harmonic Generation Fitting

The induced second‐order polarization ***P*** (2ω) is given by

(1) $$P_{i}(2\omega )\propto \chi_{ijk}^{\left(2\right)}E_{j}(\omega )E_{k}(\omega )$$

where ω is the frequency of the incoming light, 𝜒^(2)^ is the second‐order electrical susceptibility, and ***E*** (ω) is the electric field of the laser pulse.

Using the plane‐wave approximation at the sample surface (*z* = 0), the electric field propagating along the *z*‐direction, that is, along the sample surface normal, is described by

(2) $$E(k,\omega )=E_{0}(\omega )\;\cdot \;\left(\begin{array}{c} \sin\;\varphi \\ \cos\;\varphi \\ 0 \end{array}\right)$$

where φ is the angle between the electric field of the linearly polarized light pulse and the *y*‐axis and *E*_0_ is the electric field magnitude. The *xy*‐plane describes the sample surface, and the *x*‐ and *y*‐axis corresponded to the crystallographic axis [001]_o_ and ${\left[1\bar{1}0\right]}_{o}$ of the DyScO_3_ substrate, respectively. As only the leading electric‐dipole contribution to the SHG source term was considered, the independent non‐zero second‐order susceptibility components $\chi_{ijk}^{\left(2\right)}$ for the system with a macroscopic point group symmetry *m* and a *yz* mirror plane are

(3) $$\chi_{xxz}^{\left(2\right)},\;\chi_{xxy}^{\left(2\right)},\;\chi_{yxx}^{\left(2\right)},\;\chi_{yyy}^{\left(2\right)},\;\chi_{yzz}^{\left(2\right)},\;\chi_{yyz}^{\left(2\right)},\;\chi_{zxx}^{\left(2\right)},\;\chi_{zyy}^{\left(2\right)},\;\chi_{zzz}^{\left(2\right)},\;\chi_{zzy}^{\left(2\right)}$$

The inclusion of a $\chi_{xxx}^{\left(2\right)}$ component to fit the SHG signal of the as‐grown La_0.15_Bi_0.85_FeO_3_ therefore constitutes a reduction in macroscopic symmetry.

Note that all χ^(2)^‐components that included a *z*‐component of either the fundamental or the SHG light cannot be addressed as we are working in a normal‐incidence geometry. Further, all χ^(2)^‐components were chosen as complex numbers.

Finally, the observed second‐harmonic intensity ***I*** (2ω) is described by

(4) $$I(2\omega )\propto {\left|P(2\omega )\right|}^{2}$$

For further information we refer to the work in ref. [14].

### Piezoresponse Force Microscopy

The PFM measurements and the electric‐field tip poling were performed using a NT‐MDT scanning probe microscope. For scanning, a 3‐V peak‐to‐peak AC modulation was applied at 69 kHz. In order to pole the ferroelectric polarization, a DC bias was applied to the tip and the bottom SrRuO_3_ electrode was grounded. Both procedures were conducted with μmasch HQ:NSC35/Pt tips raster scanning over the surface in contact mode.

The PFM images were recorded simultaneously in Cartesian coordinates (using *X* and *Y* outputs of the lock‐in amplifiers, rather than *R* and θ). In this way, no polarization information was lost and instrumental background piezoresponse interfering with the measurements was minimized.^[^ ^36^, ^37^ ^]^

### Ferroelectric Hysteresis Loops and Fatigue Tests

Evaluation of the functionality of the devices was performed with a home‐built ferroelectric test system. The PUND technique was used for all of the measurements. In this pulse train, a preset voltage pulse of negative polarity sets the polarization. Subsequently, two pulses of positive polarity followed by two pulses of negative polarity were applied. During voltage application, the current between the top and the bottom electrode was measured. In order to avoid circuit‐related contributions, the current transients obtained from the non‐switching pulses were subtracted from the current transients obtained from the switching pulses.

To obtain hysteresis loops, triangular pulses were used and the indefinite integral was derived. During ferroelectric fatigue tests, square pulses were applied and the definite integral was used to obtain the saturation polarization.

### Training Protocol

The electric‐field training was performed with 200 rectangular voltage pulses of alternating polarity. The electric field applied was 600 kV cm^−1^, and the pulse duration as well as the relaxation time in between two voltage pulses were 100 μs.

## Conflict of Interest

The authors declare no conflict of interest.

### Author Contributions

M.M. designed and performed the experiments and the data analysis. Y.‐L.H. and R.R. provided the samples. S.V. performed photolithography and reactive ion etching. M.M., M.T., and M.F. wrote the manuscript with input from all coauthors. M.T. supervised the project jointly with M.F.

## Supporting information

Supporting Information

## Data Availability

The data that support the findings of this study are available from the corresponding author upon reasonable request.
