# Supplementary material for: Training the Polarization in Integrated La0.15Bi0.85FeO3‐Based Devices
Source: Adv Mater. 2021 Oct 24;33(52):2104688. doi: 10.1002/adma.202104688 (PMC11468450; doi:10.1002/adma.202104688)
Supplement: Supplementary file 1 — Supporting Information [file ADMA-33-2104688-s001.pdf]

ADVANCED MATERIALS

Supporting Information

for *Adv. Mater.,* DOI: 10.1002/adma.202104688

Training the Polarization in Integrated $La_{0.15}Bi_{0.85}FeO_3$-Based Devices

Marvin Müller, Yen-Lin Huang, Saül Vélez, Ramamoorthy Ramesh, Manfred Fiebig, and Morgan Trassin**

Supporting Information: Training the Polarization in Integrated $La_{0.15}Bi_{0.85}FeO_3$-Based Devices

Marvin Müller Yen-Lin Huang Saül Vélez Ramamoorthy Ramesh Manfred Fiebig Morgan Trassin*

M. Müller, Dr. S. Vélez, Prof. M. Fiebig, Dr. M. Trassin
Department of Materials, ETH Zurich, 8093 Zurich, Switzerland
marvin.mueller@mat.ethz.ch
Dr. S. Vélez
Present address: Condensed Matter Physics Center (IFIMAC) and Departamento de Física de la Materia Condensada, Universidad Autónoma de Madrid, E-28049 Madrid, Spain.
Dr. Y.-L. Huang, Prof. R. Ramesh
Department of Materials Science and Engineering, University of California, Berkeley, CA 94720, USA
Dr. Y.-L. Huang, Prof. R. Ramesh
Materials Sciences Division, Lawrence Berkeley Laboratory, CA 94720, USA
Prof. R. Ramesh
Department of Physics, University of California, Berkeley, CA 94720, USA

1 Derivation of $\boldsymbol{P}_{\text{net}}^{\text{IP}}$

The polarization of $BiFeO_3$ projected along $[001]_{\text{p.c.}}$ is 60 μC/cm².[1, 2, 3, 4] As it is oriented along the pseudo-cubic ⟨111⟩ direction, the polarization magnitude can be calculated as

$$\boldsymbol{P}_{[111]}(\text{BiFeO}_3) = \sqrt{3 \cdot \boldsymbol{P}_{[001]}^2} = 104\ \mu\text{C/cm}^2. \tag{1}$$

The single-domain in-plane polarization $P_{[110]}(\text{BiFeO}_3)$ is

$$\boldsymbol{P}_{[110]}(\text{BiFeO}_3) = \sqrt{2 \cdot \boldsymbol{P}_{[001]}(\text{BiFeO}_3)^2} = 85\ \mu\text{C/cm}^2. \tag{2}$$

$\boldsymbol{P}_{\text{net}}^{\text{IP}}$ consists of a superposition of two domains with their in-plane polarizations rotated by 90°. We therefore derive it as

$$\boldsymbol{P}_{\text{net}}^{\text{IP}} = \boldsymbol{P}_{[100]}(\text{BiFeO}_3) = \frac{\sqrt{2 \cdot \boldsymbol{P}_{[110]}(\text{BiFeO}_3)^2}}{2} = 60\ \mu\text{C/cm}^2. \tag{3}$$

2 SHG fit parameters

The non-zero parameters used for our SHG fits are displayed in Table 1. The obtained $\chi^{(2)}$-components are normalized to their maximum in order to compare their contribution to the tensor among the different samples and regions while omitting the need to account for differences in the total SHG yield. Optical SHG analysis on $BiFeO_3$ has been reported previously.[5] Note that possible strain-induced domain wall contributions were not identified.[6]

Table S1: Non-zero $\chi^{(2)}$-components obtained from our fits of the SHG anisotropy measurements in Figures 1,2 and 3. The $\chi^{(2)}_{xxx}$-component (highlighted row) is supposed to be 0 for the point-group symmetry m.[7]

$\chi^{(2)}$-component	$BiFeO_3$ pristine		$La_{0.15}Bi_{0.85}FeO_3$ pristine		$La_{0.15}Bi_{0.85}FeO_3$ poled		$La_{0.15}Bi_{0.85}FeO_3$ poled capacitor	
	Magnitude	Phase	Magnitude	Phase	Magnitude	Phase	Magnitude	Phase
xxx	0	0	0.20	$\pi/2$	0	0	0	0
xxy	0.66	0	0.48	$\pi/2$	0.48	$\pi/4$	0.48	0
yyy	1	π	1	$3\pi/4$	1	$3\pi/4$	1	$3\pi/4$
yxx	0.41	0	0.38	0	0.3	0	0.29	$\pi/2$

3 Scanning-probe microscopy of $BiFeO_3$ and $La_{0.15}Bi_{0.85}FeO_3$

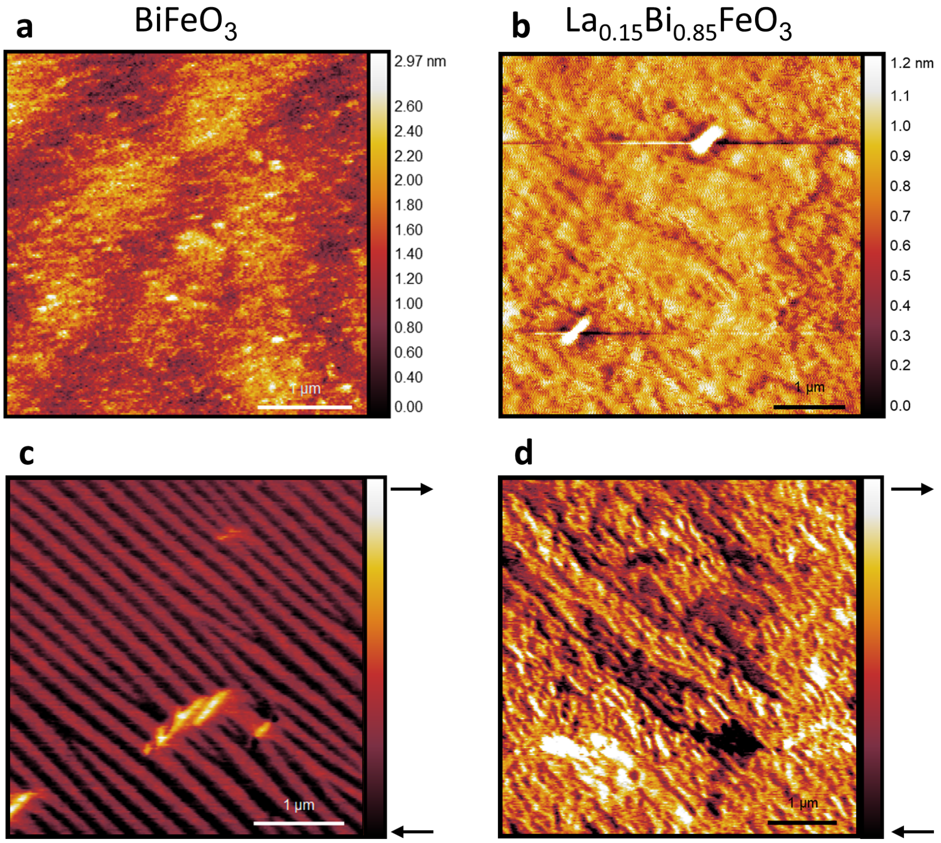

Figure S1: Same-scale AFM (**a**, **b**) and lateral-PFM (**c**, **d**) images of $BiFeO_3$ (**a**, **c**) and $La_{0.15}Bi_{0.85}FeO_3$ (**b**, **d**).

4 Negatively poled state

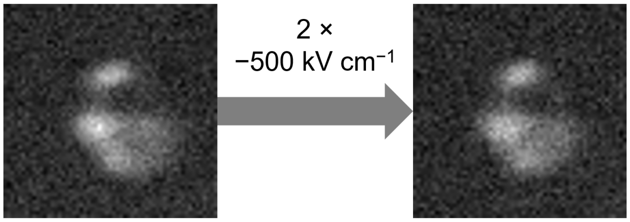

Figure S2: SHG images of the same capacitor in positively (left) and negatively (right) poled state.

References

[1] J.-G. Park, M. D. Le, J. Jeong, S. Lee, *Journal of Physics: Condensed Matter* **2014**, *26*, 43 433202.

[2] J. Wang, J. B. Neaton, H. Zheng, V. Nagarajan, S. B. Ogale, B. Liu, D. Viehland, V. Vaithyanathan, D. G. Schlom, U. V. Waghmare, N. A. Spaldin, K. M. Rabe, M. Wuttig, R. Ramesh, *Science* **2003**, *299*, 5613 1719.

[3] Y. Yang, I. C. Infante, B. Dkhil, L. Bellaiche, *Comptes Rendus Physique* **2015**, *16*, 2 193.

[4] D. Sando, A. Barthélémy, M. Bibes, *Journal of Physics: Condensed Matter* **2014**, *26*, 47 473201.

[5] M. Trassin, G. D. Luca, S. Manz, M. Fiebig, *Advanced Materials* **2015**, *27*, 33 4871.

[6] P. Marton, I. Rychetsky, J. Hlinka, *Physical Review B - Condensed Matter and Materials Physics* **2010**, *81*, 14 144125.

[7] R. R. Birss, *Symmetry and Magnetism*, North-Holland, Amsterdam, **1966**.
